# Cellular adaptations to hypoxia and acidosis during somatic evolution of breast cancer

**DOI:** 10.1038/sj.bjc.6603922

**Published:** 2007-08-07

**Authors:** R A Gatenby, K Smallbone, P K Maini, F Rose, J Averill, R B Nagle, L Worrall, R J Gillies

**Affiliations:** 1 Department of Radiology, University of Arizona, Tucson, AR 85724, USA; 2 Department of Mathematics, Oxford University, Oxford OX1 3LB, UK; 3 School of Pharmacy, Centre for Biomolecular Sciences, University of Nottingham, Nottingham NG7 2RD, UK; 4 Department of Pathology, University of Arizona, Tucson, AR 85724, USA

**Keywords:** carcinogenesis, aerobic glycolysis, GLUT-1, NHE, hypoxia, mathematical models

## Abstract

Conceptual models of carcinogenesis typically consist of an evolutionary sequence of heritable changes in genes controlling proliferation, apoptosis, and senescence. We propose that these steps are necessary but not sufficient to produce invasive breast cancer because intraductal tumour growth is also constrained by hypoxia and acidosis that develop as cells proliferate into the lumen and away from the underlying vessels. This requires evolution of glycolytic and acid-resistant phenotypes that, we hypothesise, is critical for emergence of invasive cancer. Mathematical models demonstrate severe hypoxia and acidosis in regions of intraductal tumours more than 100 *μ*m from the basement membrane. Subsequent evolution of glycolytic and acid-resistant phenotypes leads to invasive proliferation. Multicellular spheroids recapitulating ductal carcinoma *in situ* (DCIS) microenvironmental conditions demonstrate upregulated glucose transporter 1 (GLUT1) as adaptation to hypoxia followed by growth into normoxic regions in qualitative agreement with model predictions. Clinical specimens of DCIS exhibit periluminal distribution of GLUT-1 and Na^+^/H^+^ exchanger (NHE) indicating transcriptional activation by hypoxia and clusters of the same phenotype in the peripheral, presumably normoxic regions similar to the pattern predicted by the models and observed in spheroids. Upregulated GLUT-1 and NHE-1 were observed in microinvasive foci and adjacent intraductal cells. Adaptation to hypoxia and acidosis may represent key events in transition from *in situ* to invasive cancer.

Carcinogenesis is a time-dependent progression of cellular populations through increasingly disordered phenotypes culminating in emergence of an invasive cancer. This is often described as ‘somatic evolution’ because it requires a sequence of heritable genetic changes. Classical models of carcinogenesis, such as the Fearon–Vogelstein diagram (Fearon and Vogelstein, 1990), consist of accumulating alterations in oncogenes, protooncogenes, tumour suppressor genes, and senescence pathways that increase growth-promoting signals and decrease growth inhibitors.

We have proposed (Gatenby and Gillies, 2004) that traditional conceptual models do not completely capture the underlying Darwinian dynamics because they implicitly assume that the selective pressures are internal to the cells themselves, for example resistance to apoptosis. Hence, they rarely define the specific microenvironmental selection forces and their dynamical interactions with cellular phenotypic properties, which is a fundamental requirement of a complete model. This led to application of formal evolutionary mathematical models to carcinogenesis in an effort to define specific environmental selection forces and their interactions with a range of cellular phenotypes. Model simulations demonstrated a previously unknown era of carcinogenesis dominated by hypoxia (Gatenby and Vincent, 2003; Gatenby *et al*, 2005). This has led to the hypothesis that adaptation of hypoxia and acidosis are necessary steps in the later phase of the carcinogenesis sequence (Figure 1).

The proposal is based on recognition that somatic evolution of epithelial cancers occurs entirely within a space contained by a basement membrane. This anatomic constraint results in separation of the evolving tumour cells from the underlying stroma including blood vessels so that carcinogenesis occurs in an avascular environment. This enforces diffusion-reaction kinetics that limits substrate delivery to and metabolite removal from tumour cells as proliferation carries them progressively into the lumen. As a result, tumour cell proliferation even following multiple oncogene and tumour suppressor gene mutations may be limited by regional variations in oxygen, glucose, and H^+^. In the proposed carcinogenesis model, cellular adaptations to these environmental selection forces drive additional somatic evolution that is necessary for formation of an invasive cancer. Specifically, regional hypoxia promotes upregulation of glycolysis to maintain ATP production despite hypoxia, and acidosis promotes upregulation of Na^+^/H^+^ exchangers (NHEs) or mutations in apoptotic pathways to reduce acid-mediated toxicity (Park *et al*, 1999; Williams *et al*, 1999).

We propose that the final outcome of this evolutionary sequence is a population with constitutive upregulation of glycolysis and the ability to survive and proliferate in acidic conditions that would ordinarily induce quiescence or death. This constitutes a significant adaptive advantage because the phenotype creates an acidic environment (through upregulation of glycolysis) that is toxic to its competitors but less so to itself. We propose that this adaptive advantage promotes clonal expansion and invasive behaviour and that these adaptations are *necessary* for evolution of invasive epithelial cancers. The legacy of these evolutionary events is aerobic glycolysis (Warburg, 1930; Cherk *et al*, 2006) manifested by increased glucose uptake observed in FdG PET (F^18^-Fluorodeoxy-d-glucose positron emission tomography) scans in the overwhelming majority of primary and metastatic human cancers (Czernin and Phelps, 2002; Gambhir, 2002).

This conceptual model is supported by experimental observations of upregulation of cellular responses to hypoxia in regions of DCIS most distant from the basement membrane. This includes upregulation of hypoxia-inducible factor (HIF) and related proteins such as carbonic anhydrase IX and GLUT-1 (Wykoff *et al*, 2001; Brown *et al*, 2002). Furthermore, *in vivo* studies with FdG PET have demonstrated the transition from DCIS to invasive breast cancer and colonic polyps to invasive colon cancers are invariably associated with a marked increase in tumour glucose uptake (Yasuda *et al*, 2001; Abbey *et al*, 2005).

Explicit testing of the hypothesis in breast cancer through continuous observation of premalignant lesions *in vivo* is not feasible. Therefore, we initially examined our hypothesis using mathematical models to reproduce the nonlinear intra- and extracellular dynamics. Modelling simulations were then compared to cellular evolution in hypoxia and acidic regions of large (5–10 mm in diameter) multicellular spheroids maintained in culture for 30 days. The diffusion reaction kinetics of spheroids mimics those of intraductal tumour. To detect evolution of constitutive upregulation of glycolysis, we stained specimens for GLUT-1 since prior studies have demonstrated a direct correlation between GLUT-1 expression and increased glucose flux as measured by FdG PET (Brown *et al*, 1996, 2002). The presence of regional acidosis was defined by increased expression of NHE-1, which is primarily responsible for extrusion of H^+^ ions in MCF-7 and other breast cancer-derived populations (Bischof *et al*, 1996; Turturro *et al*, 2004). Finally, we examined clinical specimens of DCIS and invasive cancer to detect evidence for cellular response to hypoxia and acidosis similar to those predicted in the mathematical models and observed in the spheroids.

## MATERIALS AND METHODS

### Mathematical modelling

A hybrid cellular automaton model (Patel *et al*, 2001; Anderson, 2005; Smallbone *et al*, 2005) is used to simulate carcinogenesis on a mucosal surface in which the mutant cells are separated from the blood supply by an intact basement membrane. This two-dimensional model is composed of an array of automaton elements with a specific rule-set governing their evolution, as well as oxygen, glucose, and H^+^ fields, each satisfying reaction-diffusion equations. Each automaton element corresponds to a normal cell, tumour cell, or a vacant space. Although normal and tumour epithelial cell diameter is variable, we assume each automaton element, and hence each tumour cell has constant diameter of 25 *μ*m. A detailed description of the mathematical methods has been published (Smallbone *et al*, 2006). The mathematically inclined reader is referred to that publication and the Supplementary Appendix for a full accounting of these results.

Briefly, the model reproduces the geometry of epithelial surfaces by assuming that the bottom edge of the array represents the basement membrane. Beyond the membrane we assume the stroma is sufficiently well-vascularised such that oxygen and glucose concentrations as well as pH_e_ remain fixed at their normal extracellular values.

Initially, the automaton consists of a layer of a normal epithelial tissue and is vacant elsewhere. We assume it to be normal epithelium growing in a monolayer along the basement membrane. Cellular metabolism in the normal epithelial layer is aerobic since the cell layer possesses a normal oxygen concentration. Similarly local pH_e_ is physiologic (i.e. 7.4). Cells will undergo proliferation and death in response to microenvironmental and intracellular conditions. If oxygen and glucose concentrations are too low, then the cellular production of ATP drops below maintenance requirements and the cell undergoes apoptosis. Similarly, cells undergo apoptosis in response to an acidic pH_e_ or randomly due to senescence or loss of contact with the basement membrane. Conversely, cells that have sufficient ATP (and an adjacent site is unoccupied) may proliferate to fill that site.

To initiate carcinogenesis, we assume that the cells may begin to randomly undergo three possible heritable changes through mutations or epigenetic changes. Possible cellular phenotypic properties include:

#### Normal

These cells proliferate under normal tissue controls so that, for example, they undergo death from senescence and apoptosis when no longer in contact with the basement membrane. Thus, cellular proliferation is limited to replacement of sites that are vacant due to cell death. This phenotype typically uses aerobic metabolism of glucose but may use anaerobic glucose metabolism so that ATP production continues (although at a lesser rate) under hypoxic conditions. The population has limited tolerance for acidic pH_e_ and is assumed to undergo apoptosis at pH_e_ of less than 6.8.

#### Proliferative

This phenotype is released from normal tissue growth constraints. This is meant to mimic mutations in oncogenes, senescent pathways, and tumour suppressor genes in conventional carcinogenesis models. It allows proliferation even when not in contact with the basement membrane (i.e. anoikis-resistance) and increased crowding (i.e. resistance to contact inhibition). For purposes of analysis, we assume that these changes do not alter metabolism or the toxic effects of hypoxia or acidosis; we acknowledge, however, that mutations in some genes (such as *c*-myc) will have dual effects on both proliferation and cellular metabolism.

#### Glycolytic

These cells have constitutive upregulation of glycolysis with consequent increase in glucose uptake and acid production under all conditions including normoxia.

#### Acid-resistant

These cells proliferate under local pH_e_ conditions that would produce apoptosis in normal cells. Mechanisms for increased tolerance may include mutations in apoptotic pathways (such as caspase or p53) or upregulation of NHE-1.

These mutations give rise to eight non-normal phenotype combinations, and thus eight competing cellular populations and multiple potential evolutionary pathways.

### Tumour spheroids

We tested the predictions of the model using large tumour spheroids that recapitulate the diffusion reaction dynamics of substrate delivery and metabolite excretion. By maintaining the spheroids in microgravity (Doolin *et al*, 1999; Clejan *et al*, 2001; Dabos *et al*, 2001), we find they reliably achieve diameters of 5–10 mm. This size has three advantages: (1) it is similar to the diameter of many clinical lobular and ductal carcinoma *in situ* tumours; (2) it is much greater than the predicted diffusion limit of oxygen so that central regions of hypoxia and acidosis will develop; (3) it contains a larger number of cells than conventional spheroids, increasing the probability that evolution driven by rare mutations will be manifested during the 30-day observation period.

To observe specific evolutionary patterns predicted by the mathematical models, we studied MCF-7 cell line because it possesses two of the three heritable attributes used in the mathematical models: (1) it is hyperproliferative (e.g. it exhibits anchorage independent growth (Zugmaier *et al*, 1991)) and (2) it is resistant to acid-induced toxicity such that it remains proliferative in culture conditions even at pH of 6.5 (Figure 2). Under normal culture conditions, however, the MCF-7 cells do not exhibit constitutively upregulated glycolysis (Figure 2). Because of these phenotypic properties, we anticipate that under hypoxic and acidic conditions, MCF-7 cells will evolve phenotypes with constitutive upregulation of glycolysis, and that growth of these populations will exhibit a distinctive pattern predicted by model simulations (Figure 2, lower row) with focal nodules of glycolytic cells growing in the normoxic region of the spheroid. Finally, MUC-1 stains of MCF-7 spheroids demonstrated a basement membrane-like structure around the outer edge, further recapitulating ductal anatomy.

The spheroids were formed as follows: Confluent cell monolayers were incubated with 0.25% (*w/v*) trypsin (Sigma, UK) and 0.02% (*w/v*) EDTA (Sigma, UK) for 4 min at 37°C. Complete media were added to this mixture to inhibit enzyme activity. The cell suspension was passed through a 24-gauge needle, six times to ensure a single cell population. A 10 ml High Aspect Ratio Vessel (HARV; Synthecon, Houston, TX, USA) was seeded at 2 × 10^6^ cells ml^−1^ in a Rotary Cell Culture System (RCCS, Austin, TX, USA) rotated at 15 r.p.m. at 37°C in a humidified atmosphere of 5% CO_2_ in air. The rotation speed allowed the formed aggregates to remain stationary relative to the observer. The media in the HARVs was replenished 50 : 50 every other day. A constant concentration gradient was obtained by continuous stirring of the media.

Spheroids were removed 1, 15, and 30 days after initiation. Each spheroid was fixed and stained for GLUT-1 and NHE-1.

### Immunohistochemistry

Immunohistochemical staining for glucose transporter GLUT-1 was performed using rabbit polyclonal antibody against the C-terminal portion (Abcam, inc., Cambridge, MA, USA). The NHE-1 antibody is a rabbit polyclonal antibody (Santa Cruz Biotechnology, inc., Santa Cruz, CA, USA).

The formalin fixed, paraffin embedded spheroids were sectioned at 3 *μ*m and baked for one hour at 60°C. Immunohistochemistry was performed using the Discovery XT Automated Staining platform (Ventana Medical Systems, Inc, Tucson, AR, USA). Deparaffinisation and antigen retrieval of tissue sections was performed online. Antigen retrieval was performed on the Ventana instrument with a borate buffer (pH 8) 40 min at 95°C. Once the tissue is conditioned in this way, several blocking steps occur on the instrument. Primary antibody is applied by hand at a 1 : 800 dilution. (60 min incubation at 37°C). Primary antibodies were visualised using VMSI validated detection and counterstaining reagents. Images were captured using an Olympus BX50 camera with an RT SPOT (Diagnostic Instruments, Inc., Sterling Heights, MI, USA). Images were standardised for light intensity. Staining was performed with primary antibody in the spheroids and clinical specimens as negative controls. In GLUT-1 staining of clinical specimens, red blood cells were used as positive controls.

### Clinical specimens

Pathological specimens from 20 breast biopsies containing DCIS were subjected to immunohistochemical stains with IRB approval. Of these, five also contained regions of microscopic or gross invasive cancer. Eight of the specimens were also stained for NHE-1. In this study, we were interested in finding evidence for phenotypic adaptation to hypoxia and acidosis rather than the mechanism (i.e. activation of HIF, myc, akt, or some other pathway). For this reason, immunohistochemical analysis for overexpression of those pathways was not included in the protocols.

## RESULTS

### Modelling

**Figure 1:**
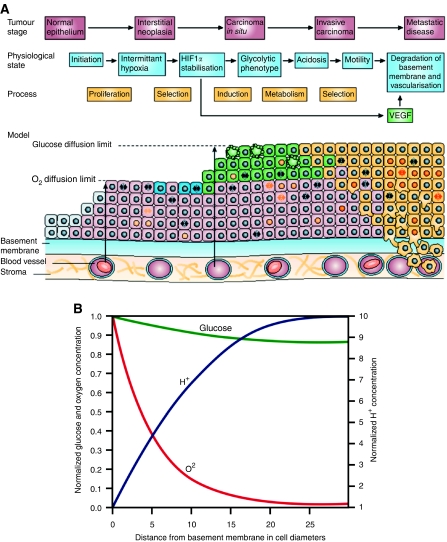
(**A**) (from Gatenby and Gillies (2004) with permission). The evolution of breast cancer over time is shown left to right. Initial heritable changes in oncogenes, tumour suppressor genes, and senescent pathways result in unconstrained proliferation into the lumen and away from the basement membrane. However, since the cells remain separated from their blood supply by the intact basement membrane, proliferation is eventually limited by hypoxia. This promotes a switch to glycolytic metabolism that eventually becomes fixed due to normoxic–hypoxic cycles. The glycolysis leads to increased extracellular acid concentrations that produces toxicity through apoptosis and necrosis. This promotes evolution of a phenotype resistant to acid-induced toxicity. This population rapidly expands because it creates an environment that (1) is toxic to other populations that remain vulnerable to acid-induced toxicity and (2) promotes invasion. We hypothesise that this allows the population to breech the basement membrane and form an invasive cancer. Note that the original model assumed central tumour necrosis was caused by a decrease in glucose concentration. The model simulation's lower panel, however, demonstrates that this component of the hypothesis is incorrect since small declines in glucose concentrations are observed. Instead, it appears that central necrosis is due to a combination of hypoxia and acidosis. (**B**) Oxygen, glucose, and H^+^ concentrations in DCIS based on diffusion reaction mathematical models; *y*-axis is normalised concentrations and *x*-axis is distance from the basement membrane in cell diameters (assuming a typical diameter of 25 *μ*m). Oxygen concentrations (red line) decline steeply with distance from the basement membrane resulting in severe hypoxia in regions only 5–10 cell layers from the membrane. Despite upregulation of glycolysis, glucose concentrations (green line) decline only modestly with distance. A steep increase in H^+^ concentrations is observed (blue line). This predicts hypoxia and acidosis will be commonly observed in ductal tumours greater than about 250 *μ*m in diameter. However, proliferation should not be limited by glucose concentrations.

The model simulation demonstrates that, dependent on oxygen consumption and cells size, significant hypoxia and acidosis will occur in DCIS within about 100 *μ*m of the basement membrane (Figure 1). This is somewhat less than the 160 *μ*m predicted by Thomlinson and Gray (1955) or shown by Helmlinger *et al* (1997) and may have been a consequence of the dimensionality. While the current simulations assumed a line source of oxygen, the above measurements were performed *in vivo* and thus oxygen could come from multiple sources.

Since 100 *μ*m represent only about five cell layers, it is clear that hypoxia will typically occur in any large (i.e. several mm in diameter) tumour-filled breast duct or lobule. This is consistent with prior studies showing increased carbonic anhydrase IX expression (indicating hypoxia and acidosis) is typically observed in central regions of DCIS (Wykoff *et al*, 2001). An unexpected finding of the simulation was the steep increase in H^+^ concentrations within the hypoxic regions. This is in distinction to the glucose concentrations that decrease only slightly. Thus, the simulations indicate that growth inhibition and ultimately necrosis in the central regions of DCIS is the result of both hypoxia and acidosis, and that reduced glucose concentration probably does not play a role. This requires a change in our original hypothesis (Gatenby and Gillies, 2004) which assumed central necrosis in DCIS was caused by limited glucose diffusion.

The simulations demonstrate that anaerobic metabolism compensates for decreased efficiency in ATP production with increased glucose flux. This adaptation, in turn, requires increased expression of membrane glucose transporters. Interestingly, we find that, even in the presence of oxygen, the anaerobic phenotype produces ATP at a slightly greater rate than aerobic metabolism. This slight advantage, however, is negated under aerobic conditions by the increased acid production that results in toxicity and, therefore, decreases cellular fitness. Under hypoxic conditions, the advantage of anaerobic metabolism greatly increases because it can maintain normal ATP production while it decreases considerably in the normal (aerobic) phenotype. Interestingly this suggests that acid-induced toxicity may serve as an important mechanism by which multicellular tissue enforces aerobic metabolism on individual cells.

**Figure 2:**
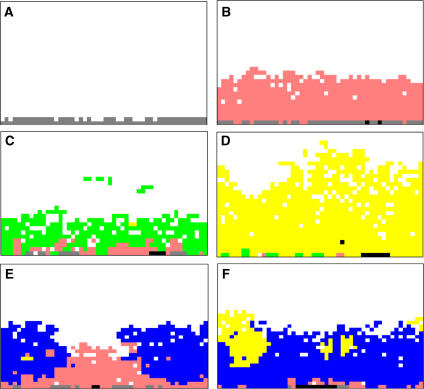
Simulations from the mathematical model described in the text showing potential evolutionary pathways in carcinoma *in situ*. Simulations start with a single layer of normal epithelial cells (grey cells) on a basement membrane (**A**). All simulations found that initial growth occurred only when mutations produced a hyperproliferative phenotype (pink cells) (**B**) through mutations in oncogenes, tumour suppressor genes, etc. Growth into the lumen eventually ceased, however, due to hypoxia and acidosis (Figure 1B). Without additional cellular evolution, this population remains limited. Additional growth occurred following two possible sequences: (1) heritable changes that upregulate glycolysis. This population with constitutive upregulation (green cells) (**C**) allow this new population to replace the hyperplastic cells and to extend further into the lumen. However, clonal expansion is eventually limited by acid-mediated toxicity. This promotes evolution of a glycolytic, acid-resistant phenotype (yellow cells) which rapidly replaces all other extant populations in a highly aggressive, infiltrative pattern extending to the basement membrane and farther into the lumen (**D**). (2) A second pathway begins with development of an acid-resistant population (blue cells). This population expands and replaces many of the hyperplastic population (**E**) but growth remains limited by hypoxia promoting emergence of a phenotype with upregulated glycolysis and acid resistance (yellow cells) identical to the population in (**C**). However, unlike in (**C**), this phenotype initially grows into the normoxic region forming nodules of varying size (**F**). These eventually coalesce into a pattern essentially identical to the appearance in (**D**).

Figure 2 shows temporal evolutionary patterns observed in model simulations. Initially, normal epithelial cells (grey) line the basement membrane. In the first stage of carcinogenesis, only those mutations that resulted in a relaxation of normal cell–cell growth constraints (pink) allowed growth. Mutations that changed glucose metabolism or acid tolerance conferred no proliferative advantage so that neither phenotypic trait was maintained in the intraductal populations.

The simulations demonstrate that alteration in growth regulators are necessary but not sufficient to form a cancer because proliferation eventually ceases as clonal expansion moves cells further into the lumen. This results in cessation of growth by hypoxia and acidosis.

**Figure 3:**
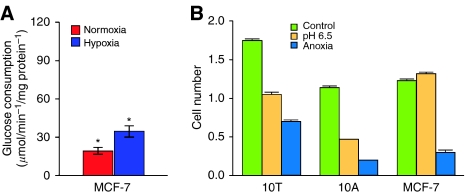
(**A**) Cultured MCF-7 cells exhibit a low level of glucose metabolism under normoxic conditions. Under hypoxic condition, MCF-7 cells increase expression of HIF-1*α* resulting in upregulation of glycolysis and increased glucose consumption. This indicates this population exhibits a normal Pasteur effect and does not, at least initially, exhibit aerobic glycolysis (Warburg effect). (**B**) Survival of MCF-7 cells compared to 10A and 10T cells (using MTT assay) after 6 days under normal, anoxic, or acidic (DMEM with 10% FBS in humidified room air with 5% CO_2_ or nitrogen at pH 7.4 or 6.5) culture conditions. Note that all of the cell lines were adversely affected by anoxia, but the MCF-7 cells continued to proliferate under extremely acidic conditions (*P*<0.01).

During multiple simulations, cellular adaptation to these environmental selection forces followed two evolutionary pathways. In one (Figure 3, middle row), the glycolytic phenotype occurs first and becomes dominant (green). This population because of its increased expression of glucose transporters and increased glycolytic capacity produces substantially more ATP in hypoxic conditions and, so, has a competitive advantage. As demonstrated in Figure 3, the glycolytic cells replace the hyperplastic population in the hypoxic tumour regions. Note, however, that the total number of cells within the system has decreased because the increased glycolysis has resulted in higher levels of acidity, in turn inducing cell death. Further adaptation occurs to an acid-resistant phenotype (Figure 3, middle right). This population expands rapidly replacing the normal, hyperplastic, and hyperplastic plus glycolytic populations and invades into the normoxic region. This is because the population continues to use glycolytic pathways and produce local acidosis even in the presence of oxygen causing all cells with normal acid tolerance to die off.

The second evolutionary pathway begins with emergence of the acid-resistant phenotype (blue cells in Figure 2 lower row). This allows proliferation in the acidic regions (which are also hypoxic), thus, confers a proliferative advantage permitting population expansion. A subsequent mutation that increases glycolysis generates more ATP allowing this population to expand initially in the hypoxic region. However, this phenotype appears to have only a modest adaptive advantage compared to the glycolytic, proliferative phenotype. This produces slow population growth in a distinctive nodular pattern as demonstrated in Figure 3 (lower right panel).

### Spheroids

**Figure 4:**
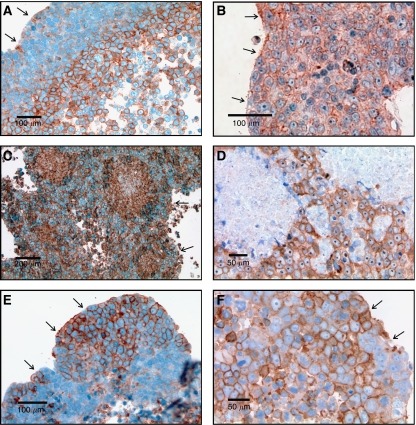
Multiple immunohistochemistry images from spheroid at 1, 15, and 30 days following formation. The arrows mark the outer (oxygenated) edge of the spheroid. (**A**) Demonstrates GLUT-1 antibody distribution at day 1 with upregulation confined to the central regions of the spheroids presumably in response to hypoxia. (**B**) Shows NHE-1 staining at day 1 with uniform expression in both peripheral and central regions. This is consistent with constitutive upregulation as predicted by MCF-7 resistance to acidosis (Figure 3B). Uniform distribution of NHE-1 remained on day 15 and 30 spheroids. (**C**) Demonstrates GLUT1 expression in a spheroid at day 15 showing multiple nodules of cells with increased GLUT 1 throughout the rim similar to the nodular morphology predicted by the mathematical simulation in Figure 2F demonstrates. Note the nodule on the right contains central necrosis. (**D**) Shows NHE-1 distribution in the same spheroid in (**C**). Note that NHE-1 expression remains uniform throughout the specimen even adjacent to the areas of necrosis consistent with constitutive upregulation of NHE-1 expression. (**E**) Demonstrates GLUT1 distribution in a spheroid on day 30 which shows a cluster of cells expressing with increased expression of GLUT 1 in the peripheral, normoxic region of the spheroid. (**F**) A high magnification from the spheroid in (**E**) showing upregulation of GLUT-1 is primarily in the membrane but with increased cytoplasmic expression in some cells.

All three spheroids harvested at day 1 exhibited evidence of hypoxia in the core with upregulation of GLUT-1 in cells about 100–50 *μ*m from the edge representing an adaptation to hypoxia (Figure 4). Staining for NHE-1 was observed throughout all of the cells of the spheroid at all time points so that there was no evidence of regional or temporal variations in NHE-1 (Figure 4). In two (of three) spheroids harvested at day 15 following initiation and two (of three) spheroids harvested at day 30, cells exhibiting increased GLUT-1 were observed in the periphery of the spheroid (Figure 4) which is normoxic base on both the simulations and the absence of increased GLUT 1 expression on the spheroids at day 1. In each spheroid, the nodules varied in size – a pattern remarkably similar to the model simulations. GLUT-1 and HIF-1*α* were co-expressed (not shown) indicating that increased glycolysis was regulated by stabilisation of HIF-1*α*.

### Clinical specimens

**Figure 5:**
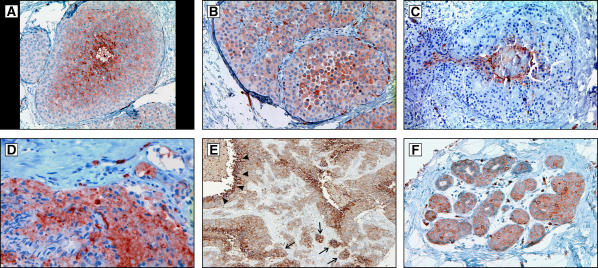
Examples of GLUT-1 distribution in DCIS and invasive breast cancer. (**A**) Shows central distribution of upregulated GLUT-1 with a gradient of intensity that parallels the transition from normoxia to hypoxia as predicted in Figure 1B and similar to the gradient observed in spheroids at day 1 (Figure 4A). (**B**) Demonstrates a nodule of cells that predominantly demonstrate upregulation of GLUT-1 in the periphery of DCIS similar to the nodules seen in spheroids (Figure 4C and D). (**C**) Demonstrates extension of cells with upregulated GLUT-1 from the periluminal regions directly into a focus of invasion. (**D**) Show populations of cells with increased GLUT-1 expression in the periphery of DCIS adjacent to foci of microinvasion in which the cells also have increased GLUT-1 expression. Note the diffuse intracellular staining (i.e. membrane, cytoplasmic, and nuclear). This pattern has been noted in a prior study (Brown *et al*, 2002). (**E**) Demonstrates a region of DCIS in the upper left increased GLUT-1 expression only in the luminal, hypoxic cells (arrowheads). In the lower right are foci of microinvasion with increased GLUT-1 expression (arrows). Note the cells with increased GLUT-1 expression adjacent to the basement membrane in the adjacent tumour filled duct. (**F**) Demonstrates GLUT-1-positive cells in an invasive cancer.

Twenty clinical specimens with DCIS were reviewed for evidence of cellular adaptations predicted by the mathematical model and observed in spheroids. In all but one of the samples, tumour cells with upregulated GLUT-1 were observed in at least some regions of DCIS. In all 19 samples, increased GLUT-1 expression was present in the central regions of intraductal tumours (Figure 5). In 17 of 19 specimens cells with upregulated GLUT-1 were also observed in the peripheral (presumably normoxic regions) of some of the intraductal tumours. In all of these cases, the cells formed clusters similar in pattern to the model simulations and spheroids suggesting that adaptation to acidosis typically precedes constitutive upregulation of glycolysis. This differs from the modelling results in which upregulated glycolysis preceded development of acid resistance in most simulation. The reason for this will be the subject of further study.

In four specimens, a focus of microinvasive tumour was observed adjacent to a tumour-filled duct. In each of these cases, the cells in the invasive tumour demonstrated upregulated GLUT-1 as did the cells in the periphery of the DCIS immediately adjacent to the focus of microinvasion. Upregulation of GLUT-1 was observed in cells within four of five invasive cancers (Figure 5). The cellular expression of GLUT-1 was often both membranous and cytoplasmic – a pattern previously observed (Brown *et al*, 2002).

**Figure 6:**
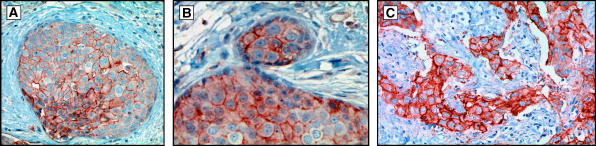
(**A**) Shows NHE-1 distribution in DCIS. In much of the lesion, NHE-1 expression is greatest in the central hypoxic region. Some cells with strongly increased NHE-1 expression, however, are extending into the normoxic region where there is a focal bulge into the basement membrane. In (**B**), there is a population of cells exhibiting increased NHE-1 expression in the periphery of DCIS adjacent to a focus of microinvasion which also exhibits increased expression of NHE-1. (**C**) Demonstrates upregulated NHE-1 in cells within an invasive breast cancer.

Regions of cells with increased expression of NHE-1 were observed in all eight specimens examined (Figure 6). In DCIS, the distribution typically showed areas of increased expression both centrally and in the periphery. Distinctive nodules such as those seen in GLUT-1 distribution were not observed. In the four cases of microinvasion, upregulated NHE1 expression was observed both in the invasive cells and in the DCIS cells immediately adjacent to the foci of microinvasion. In all three cases of invasive cancer examined, NHE-1 was upregulated diffusely in the tumour cells.

## DISCUSSION

We propose regional variations in substrate and metabolite concentrations in DCIS initiate cellular adaptations necessary for emergence of invasive breast cancer (Gatenby and Gillies, 2004). The hypothesis is framed mathematically using a modified cellular automata approach. The reaction–diffusion simulations support a key element of the hypothesis by demonstrating severe hypoxia within about five cell layers of the basement membrane in DCIS. However, the results do not support the original hypothesis that a decrease in glucose concentrations leads to central necrosis. Rather, we find that glucose concentrations decline only mildly with distance from the basement membrane while acid concentrations increase more than originally expected. The results indicate necrosis observed within DCIS is likely due to hypoxia and acidosis rather than to insufficient glucose supply.

Model simulations demonstrate that different evolutionary trajectories may occur in adaptation to hypoxia and acidosis but will generally converge to a final phenotype with constitutive upregulation of glycolysis and resistance to acid-induced toxicity. This phenotype invades into the normoxic regions of the premalignant lesion because it creates an acidic environment through upregulated glycolysis that is toxic to other cellular populations, which remain vulnerable to acid-mediated toxicity (Park *et al*, 1999; Williams *et al*, 1999; Gatenby and Gillies, 2004).

We propose this evolutionary sequence is critical in late carcinogenesis because it confers a proliferative advantage on the population and enhances invasiveness (Rohzin *et al*, 1994; Cuvier *et al*, 1997). The latter is necessary for breeching the basement membrane and transition to invasive cancer.

The model simulations predict a distinctive pattern of nodular growth when populations with constitutively increased glycolysis emerge following mutations in a dominant population that is acid resistant.

We examined this prediction by observing the evolutionary dynamics of MCF-7 cells growing in spheroids under microgravity conditions. The spheroids, which measure 5–10 mm in diameter, mimic the diffusion reaction kinetics in DCIS. At baseline, the MCF-7 cells are resistant to acid-induced toxicity but do not exhibit constitutive upregulation of glycolysis. We demonstrate evolutionary dynamics in which upregulation of GLUT-1 is initially observed only as an adaptation to hypoxia. Over time, populations of GLUT-1-positive cells are observed invading into the peripheral normoxic regions of the spheroids. The proliferation of clones of cells with upregulated GLUT-1 under physiologic conditions is consistent with our hypothesis that this phenotype confers a proliferative advantage. The nodular growth pattern is remarkably similar to the modelling predictions also supporting this hypothesis. Demonstration of a proliferative advantage to the glycolytic phenotype even under normoxic conditions provides a mechanism by which aerobic glycolysis (the Warburg effect) could arise during somatic evolution of cancer populations.

Immunohistochemical staining in DCIS demonstrated increased expression of GLUT-1 in central, hypoxic regions – a pattern also observed by Brown *et al* (2002). In addition, in 17 of 20 cases, we also observed some populations with upregulated GLUT-1 in the peripheral, normoxic regions of DCIS. Regions of increased NHE-1 expression were also observed in all of the DCIS specimens examined. The results are consistent with our hypothesis that hypoxia and acidosis occur frequently in DCIS, and that cellular adaptations confer an adaptive advantage that allows subsequent spread into regions that are normoxic with normal pH_e_.

All of the invasive cancers examined demonstrated cells with increased NHE-1 expression and four of five contained cells with upregulated GLUT-1. The latter value is slightly greater than the 61% of invasive cancers with increased GLUT-1 previously reported (Brown *et al*, 2002). These results support the hypothesis that the glycolytic, acid-resistant phenotype is important in transition from DCIS to invasive cancer. The absence of universal observation of upregulation GLUT-1 in invasive cancer may indicate that aerobic glycolysis is not always necessary for invasive phenotype. Alternatively, some breast cancer populations may maintain increased glycolysis through upregulated of GLUT-3 or use fatty acids rather than glucose as their dominant carbon source (Liu, 2006).

There are several limitations in the current study. We cannot with certainty know that the peripheral regions of the DCIS lesions are uniformly well oxygenated and, thus, it is possible that upregulation of GLUT1 and NHE-1 are adaptive (to regional hypoxia) rather than constitutive. Furthermore, the sample size is relatively small and the data descriptive and non-quantitative. Finally, we do not know if similar dynamics are seen in other premalignant lesions such as colon adenomas. However, the empirical results in combination with the mathematical modelling simulations do support the hypothesis that the hypoxia–glycolysis–acidosis cycle does occur in somatic evolution of breast cancer and that further investigation is warranted.
