# Supplementary material for: Cellular adaptations to hypoxia and acidosis during somatic evolution of breast cancer
Source: Br J Cancer. 2007 Aug 7;97(5):646–53. doi: 10.1038/sj.bjc.6603922 (PMC2360372; doi:10.1038/sj.bjc.6603922)
Supplement: Supplementary Appendix [file 6603922x1.pdf]

Appendix

Cellular metabolism

We first define a simple model of cellular glucose metabolism. Under normal physiological conditions, human cells rely on aerobic respiration to produce their energy. Each glucose molecule reacts with six oxygen molecules to produce carbon dioxide and ATP. This reaction may be caricatured by

$$glucose + 6\,O_2 \;\rightarrow\; 6\,CO_2 + n_A\,ATP$$

where n_A denotes the number of ATP produced during complete oxidation of glucose. In this paper we assume $n_A = 36$, though this value may vary slightly depending on the specific cell type under consideration.

During periods of hypoxia, cells revert to the less efficient anaerobic metabolism, producing two molecules of lactic acid per glucose

$$glucose \;\rightarrow\; 2\,lactic\,acid + 2\,ATP.$$

The details of the mathematical models capturing cellular dynamics are included in the appendix. During the first stage of carcinogenesis, the dominant growth constraints involve cellular interactions with the extracellular matrix and other cells. During this phase, substrate supplies are assumed adequate and, therefore, cellular metabolism neither promotes nor constrains growth. Once these social constraints have been overcome and tumor cells proliferate into the lumen and away from the basement membrane, the dominant growth constraint becomes limited substrate availability, and thus increased ATP production confers a competitive advantage.

The dynamics of aerobic and anaerobic metabolism of glucose are summarized in Eqs. (1)–(4). Cells using inefficient glycolytic metabolism maintain adequate ATP concentrations by increasing glucose flux. Normal cells are assumed to adopt glycolytic metabolism only when environmental conditions are hypoxic [1]. Transformed cells exhibit a similar response to hypoxia but also maintain glycolytic metabolism even in the presence of oxygen [2]. Differences between the two cell types are also seen in H^+ production. Normal cells produce increased acid (i.e. above the basal rate) only when oxygen supply is low. However, glycolytic cells produce increased amounts of H^+ even in normoxic conditions and thus acidify the extracellular space, irrespective of the oxygen levels.

Metabolite profiles

Having defined a model of cellular respiration, we are now in a position to determine the metabolite distributions around the cells. The details of the mathematical modelling are included in the appendix. After each automaton generation, the known rates of metabolite consumption and production for each cell are used to calculate the corresponding metabolite profiles. This allows us to generate a continuously varying regional map of oxygen, glucose,

and acid concentrations.

Cell Dynamics

We now proceed to investigate how the carcinoma evolves in response to the associated distribution of glucose, oxygen and H^+ within the tissue. Initially, the automaton is composed of normal cells forming a monolayer along the basement membrane. After each generation, the resultant glucose, oxygen and H^+ fields are calculated using the methods outlined above. Each cell in the automaton is then updated (in a random order) according to the local metabolite levels. Cells may proliferate, adapt or die, and cells with different phenotypic patterns respond to the microenvironmental pressures in different ways. As such, competition is incorporated into the model: for a new population to progress and grow, it must successfully compete for space and resources with existing populations.

The rules governing the evolution of the automaton elements are as follows:

1. An element that is empty does not evolve directly. It may evolve indirectly when cell division takes place in a neighboring cell.

2. If the amount of ATP produced by a cell ϕ_a falls below a critical threshold value, a_0, it dies, and the element becomes empty. As such, a_0 represents the level of ATP required for normal cellular maintenance. We do not allow hypoxia to directly induce cellular death within our model. Rather, hypoxia indirectly causes cell death through a reduction in ATP production. As mentioned previously, cells displaying the glycolytic phenotype produce significantly more ATP than their normal counterparts during periods of hypoxia, thus they are less susceptible to cell death via this mechanism. We assume $a_0 = 0.1$, corresponding to normal cell death occurring when oxygen levels drop below $c = 0.05$ (3)

3. The loc$D_G \nabla^2 G - \Phi_G = 0,$al H^+ level may also induce cellular death, with probability p_{dea}. We define this probability by

$$p_{\text{dea}} = \begin{cases} h/h_N & \text{in a normal cell, if } h < h_N, \\ h/h_T & \text{in an acid-resistant cell, if } h < h_T, \\ 1 & \text{otherwise.} \end{cases}$$

where $h_N < h_T$. Thus the probability of cell death increases with acidity, and the cell will always die if the H^+ level is greater than h_N or h_T, dependent on the cell type under consideration. These values are taken to be $h_N = 9.3 \times 10^2$ and $h_T = 8.6 \times 10^3$ for normal and acid-resistant cells, respectively, corresponding to threshold values of pH 6.8 and pH 6 (4).

4. If the cell is not attached to the basement membrane, and is not hyperplastic, it dies.

5. If the cell does not die through any of the mechanisms above, it either attempts to divide, with probability p_{div}, or becomes quiescent. The probability of division is a function of the cellular ATP production

$$p_{\text{div}} = \begin{cases} (\phi_a - a_0)/(1 - a_0) & a_0 < \phi_a < 1, \\ 1 & \phi_a \geq 1. \end{cases}$$

Hence we assume that the probability of division is proportional to the ATP generated that is not needed for maintenance, and that the cell will always attempt to divide if the production rate is more than its normal level of 1. If the cell attempts to divide, we determine whether cell division occurs by sampling its neighboring elements. If there is one empty space, then the cell divides, and the new cell occupies this empty space. If there is more than one empty space, the new cell goes to the element with the largest oxygen concentration[5].

6. If a cell divides, each of the two daughter cells has probability p_a of randomly acquiring one of the three heritable characteristics (hyperplasia, glycolysis and acid-resistance). In order to avoid bias in the model, we assume these changes are reversible. For example, a cell displaying constitutive up-regulation of glycolysis may revert to normal glucose metabolism; if this metabolism is most appropriate for the current microenvironmental conditions, the cell will successfully compete for resources with its neighbors. We choose $p_a = 10^{-3}$ as a base value, to reflect the fact that heritable change is a relatively rare occurrence.

It remains to define the dimensions of the automaton M and N. We take $N = 50$, corresponding to a typical ductal carcinoma of radius 200 μm. However, we leave M undefined, allowing it to dynamically increase as the carcinoma grows. Essentially the final value taken by M will represent the maximum distance from the basement membrane the cells may survive, given the limited nutrient supply and acid removal.

Throughout this model derivation, we have assumed that the various processes above follow simple, linear dynamics. It can be argued that these assumptions are too unrealistic to represent complex biological phenomena such as these. However, these processes are poorly understood and, as a first approximation, an assumption of linearity is sufficient to capture qualitatively similar monotonic behavior. We would not expect these assumptions to have a marked effect on the model's conclusions. Moreover, the relative simplicity of the model means that the parameter space is kept to a manageable size.

Model of cellular metabolism

Suppose the cell consumes glucose and oxygen at rates Φ_G and Φ_C, respectively, and that all of the consumed glucose and oxygen is used to generate ATP under the two processes

outlined above. Then, from above, we are assuming $\Phi_G \geq \Phi_C/6$. If this condition is satisfied, we may calculate the rates of ATP production Φ_A and lactic acid production Φ_L

$$\Phi_A = \frac{n_A \Phi_C}{6} + 2(\Phi_G - \frac{\Phi_C}{6}), \quad (1)$$

$$\Phi_L = 2(\Phi_G - \frac{\Phi_C}{6}). \quad (2)$$

The lactic acid produced by the cell partially disassociates into H^+ and lactate. These H^+ ions lower the pH of the extracellular space, inducing cellular toxicity. We caricature the rate of cellular H^+ production Φ_H as proportional to the rate of lactic acid production, $\Phi_H = k_H \Phi_L$, for some $k_H < 1$. Note that the aerobic pathway also contributes to cellular acid production through hydration of CO_2. However, this contribution is small – for each mole of ATP synthesized, anaerobic metabolism produces one mole of lactic acid, whilst aerobic metabolism produces only 1/6 mole of CO_2. As such we ignore this term, considering only the acid production in excess of the normal rate.

It remains to define the rates of cellular glucose and oxygen consumption Φ_G and Φ_C. Whilst complex empirical functional forms for these rates are available[6] here we assume that the rates follow simpler first-order dynamics

$$\Phi_G = \begin{cases} k_N G & \text{in a normal cell,} \\ k_T G & \text{in a glycolytic cell,} \end{cases} \quad (3)$$

$$\Phi_C = k_C C, \quad (4)$$

where G and C denote the extracellular concentrations of glucose and oxygen, respectively, and $k_T > k_N$. Note that we assume that tumor cells do not significantly alter their rate of oxygen consumption during carcinogenesis, consistent with experimental observations[7]

We non-dimensionalize Eqs. (1) – (4), to reduce the size of the parameter space. Let G_X and C_X denote the normal extracellular concentrations of glucose and oxygen, and suppose that under normal conditions, normal cells rely on aerobic respiration alone to produce energy. Then $k_C C_X = 6 k_N G_X$ and

$$\phi_g = \begin{cases} g & \text{in a normal cell,} \\ kg & \text{in a glycolytic cell,} \end{cases} \quad (5)$$

$$\phi_c = c, \quad (6)$$

$$\phi_a = c + n(\phi_g - c), \quad (7)$$

$$\phi_h = \phi_g - c, \quad (8)$$

subject to the condition $\phi_g \geq c$, where

$$g = \frac{G}{G_X}, c = \frac{C}{C_X}, \phi_g = \frac{\Phi_G}{k_N G_X}, \phi_c = \frac{\Phi_C}{k_C C_X}, \quad (9)$$

$$\phi_a = \frac{\Phi_A}{n_A k_N G_X}, \phi_h = \frac{\Phi_H}{2 k_H k_N G_X}, n = \frac{2}{n_A}, k = \frac{k_T}{k_N}. \quad (10)$$

The non-dimensionalized model of cellular respiration relies on two parameters: $n = 1/18$

and k. Given ranges $10^{-6}\,\mathrm{s}^{-1} < k_N < 5 \times 10^{-4}\,\mathrm{s}^{-1}$ and $10^{-5}\,\mathrm{s}^{-1} < k_T < 10^{-3}\,\mathrm{s}^{-1}$ (8) for the rates of glucose consumption by normal and tumor cells, respectively, we assume $1 < k < 10^3$, i.e. that glycolytic cells may increase their glucose consumption by up to three orders of magnitude

Metabolite Models

Consider first the extracellular concentration of glucose, G. Note that the glucose diffusion time-scale (~minutes) is much shorter than the cellular proliferation timescale (~days), and thus we may assume that G is in diffusive equilibrium at all times. Then we have

$$D_G \nabla^2 G - \Phi_G = 0, \tag{11}$$

where D_G is the (assumed constant) glucose diffusion coefficient. We non-dimensionalize Eq. (11), taking cell diameter as our length scale. Using Eq. (10),

$$d_g^2 \nabla_\xi^2 g - \phi_g = 0, \tag{12}$$

where $\xi = x/\Delta$ and $d_g = \sqrt{D_G/k_N\Delta^2}$. Given $D_G = 5 \times 10^{-6}\,\mathrm{cm}^2\,\mathrm{s}^{-1}$ (9) and taking $k_N = 5 \times 10^{-5}\,\mathrm{s}^{-1}$, we find $d_g = 1.3 \times 10^2$. In a spatially homogeneous system of normal cells, $d_g \log 2 \approx 90$ represents the number of cells away from the basement membrane at which the glucose concentration drops to half its normal level. In a system of glycolytic cells, where glucose is consumed at a higher rate, this distance falls to $d_g \log 2/\sqrt{k}$.

Eq. (12) is solved using a finite-difference approximation

$$g_{i+1,j} + g_{i-1,j} + g_{i,j+1} + g_{i,j-1} - (4 + \delta_{i,j})g_{i,j} = 0, \tag{13}$$

where $g_{i,j}$ refers to the glucose level of the *i-j*th automaton element and $\delta_{i,j}$ depends on the element's occupancy

$$\delta_{i,j} = \begin{cases} 0 & \text{in a vacant cell,} \\ 1/d_g^2 & \text{in a normal cell,} \\ k/d_g^2 & \text{in a glycolytic cell.} \end{cases} \tag{14}$$

As boundary conditions, we assume that the glucose levels are fixed at their normal levels at the basement membrane (as the stroma is well-vascularized), zero flux at the edge furthest from the membrane (as there are no sources or sinks of glucose beyond this point), and periodic boundary conditions at the other two edges. Using the notation of Eq. (13), this may be written as

$$g_{0,j} = 1, \;\; g_{M+1,j} = g_{M,j} \quad \forall j = 1, \ldots, N, \tag{15}$$

$$g_{i,0} = g_{i,N}, \;\; g_{i,N+1} = g_{i,1} \quad \forall i = 1, \ldots, M. \tag{16}$$

Eq. (13) holds $\forall i = 1, \ldots, M$ and $\forall j = 1, \ldots, N$ and is thus representative of a system of $M \times N$ linear algebraic equations in the unknowns $g_{i,j}$. The equilibrium glucose field $g = (g_{i,j})$ may then be found through simple matrix inversion.

The oxygen distribution around the tumor is found using the same method. In

non-dimensional form we have

$$d_c^2 \nabla_\xi^2 c - \phi_c = 0, \tag{17}$$

where $d_c = \sqrt{D_C / k_C \Delta^2}$ and D_C is the oxygen diffusion coefficient. Given $k_C = 9.41 \times 10^{-2}\,\mathrm{s}^{-1}$ (6) and $D_C = 1.46 \times 10^{-5}\,\mathrm{cm}^2\,\mathrm{s}^{-1}$ (10), we find $d_c = 5 \ll d_g$. In stark contrast to glucose, oxygen supply is very limited due to its small relative diffusion rate, with areas of hypoxia developing within a few cells of the basement membrane. Note that, in order for the model to be well-defined, we require $\phi_g \geq c$ at each cell, for which it is sufficient that $g \geq c$ everywhere. This holds if $k \leq d_g^2/d_c^2 \approx 700$ and as such we restrict our attention here to the parameter range $1 < k \leq 500$.

The equilibrium oxygen field c is found from Eq. (17) using the same technique as for glucose. Having determined the glucose and oxygen fields, we know their rates of consumption, ϕ_g and ϕ_c, for each individual cell. Then, from Eq. (9), we may calculate the rate of cellular H^+ production, ϕ_h. Unlike glucose and oxygen, H^+ ions do not follow simple (Fickian) diffusion, as this would lead to charge separation. Rather, they diffuse in association with mobile buffering species such as bicarbonate, phosphate, or amino acids[11]. However, their movement may be approximated by simple diffusion, with appropriate modification of the diffusion coefficient. Thus the H^+ distribution, h, is defined by

$$\nabla_\xi^2 h + \phi_h = 0, \tag{18}$$

where $h = (H - H_X)/H_0$ and $H_0 = 2k_H k_N G_X \Delta^2 / D_H$. Here H is the extracellular concentration of H^+ , $H_X \equiv pH\,7.25$ its normal level and D_H its effective diffusion coefficient. This specific form for the scaling factor H_0 is chosen to remove the diffusion coefficient from Eq. (18). Given parameter values $D_H = 1.08 \times 10^{-5}\ cm^2\ s^{-1}$ and a maximum tumor acid production rate of $10^{-4}\ mM\ s^{-1}$ (4) and assuming this is equivalent to our maximum non-dimensionalized rate of $\phi_h = 500$, we may estimate $H_0 = 1.1 \times 10^{-7}\ mM$.

Eq. (18) is solved as before using a finite-difference approximation, with the difference in this case that $h = 0$ is the normal level at the basement membrane.

References for Appendix

1. Racker, E. History of the Pasteur effect and its pathobiology. Mol Cell Biochem 1974; **5**:17-23.

2. Warburg, O. *The Metabolism of Tumours.* London: Constable Press; 1930.

3. Anderson, ARA. A hybrid mathematical model of solid tumour invasion: the importance of cell adhesion. *Math Med Biol* 2005; **22**:163–186.

4. Patel, AA, Gawlinski ,ET, Lemieux, SK, Gatenby, RA. A cellular automaton model of early tumor growth and invasion. *J Theor Biol* 2001; **213**:315–331.

5. Alarcón, T, Byrne, HM, Maini ,PK. A cellular automaton model for tumour growth in an inhomogeneous environment. *J Theor Biol* 2003; **225**:257–274

6. Casciari, JJ, Sotirchos, SV, Sutherland, RM. Variations in tumor cell growth rates and

metabolism with oxygen concentration, glucose concentration, and extracellular pH. *J Cell Physiol* 1992; **151**:386–394.

7.Ramanathan, A, Wang,C, Schreiber, SL. Perturbational profiling of a cell-line model of tumorigenesis by using metabolic measurements. *Proc Natl Acad Sci USA* 2005; **102**:5992–5997.

8. Kallinowski, F et al. Glucose uptake, lactate release, ketone body turnover, metabolic milieu and pH distributions in human cancer xenografts in nude rats. *Cancer Res* 1988; **48**:7264–7272.

9. Groebe, K, Erz,S, Mueller-Kleiser, W. Glucose diffusion coefficients determined from concentration profiles in EMT6 tumor spheroids incubated in radioactively labeled L-glucose. *Adv Exp Med Biol* 1994; **361**:619–625.

10. Nichols ,MG, Foster, TH. Oxygen diffusion and reaction kinetics in the photodynamic therapy of multicell tumour spheroids. *Phys Med Biol* 1994; **39**:2161–2181.

11.Schornack, PA, Gillies, RJ. Contributions of cell metabolism and H^+ diffusion to the acidic pH of tumors. *Neoplasia* 2003; **5**:135–145.
